# Inter-species interconnections in acid mine drainage microbial communities

**DOI:** 10.3389/fmicb.2014.00367

**Published:** 2014-07-25

**Authors:** Luis R. Comolli, Jill F. Banfield

**Affiliations:** ^1^Structural Biology and Imaging Department, Life Sciences Division, Lawrence Berkeley National LaboratoryBerkeley, CA, USA; ^2^Department of Earth and Planetary Science, University of California, BerkeleyBerkeley, CA, USA

**Keywords:** Cryo-TEM, Intact natural microbes, Archaea, Microbial inter-species connections, Metagenomics, *Thermoplasmatales*, ARMAN

## Abstract

Metagenomic studies are revolutionizing our understanding of microbes in the biosphere. They have uncovered numerous proteins of unknown function in tens of essentially unstudied lineages that lack cultivated representatives. Notably, few of these microorganisms have been visualized, and even fewer have been described ultra-structurally in their essentially intact, physiologically relevant states. Here, we present cryogenic transmission electron microscope (cryo-TEM) 2D images and 3D tomographic datasets for archaeal species from natural acid mine drainage (AMD) microbial communities. Ultrastructural findings indicate the importance of microbial interconnectedness via a range of mechanisms, including direct cytoplasmic bridges and pervasive pili. The data also suggest a variety of biological structures associated with cell-cell interfaces that lack explanation. Some may play roles in inter-species interactions. Interdependences amongst the archaea may have confounded prior isolation efforts. Overall, the findings underline knowledge gaps related to archaeal cell components and highlight the likely importance of co-evolution in shaping microbial lineages.

## Introduction

The fact that microbes shape the biosphere is well appreciated, but there are many blind spots in our understanding of the relevant processes because over half of all microbial phyla lack even a single characterized member (Baker and Dick, 2013). The tree of life is being constantly revised and expanded with the addition of new microbial genomes that have been obtained by cultivation-independent methods (Hall-Stoodley et al., 2004; Tyson et al., 2004; Fuhrman, 2009; Wrighton et al., 2012; Castelle et al., 2013; Di Rienzi et al., 2013; Kantor et al., 2013; Rinke et al., 2013). Systems-biology tools are being developed to uncover networks of interactions among microorganisms, and simulations begin to predict system-wide responses and adaptations (Dubey and Ben-Yehuda, 2011; Sanchez, 2011; Cremer et al., 2012). Yet, basic information about the nature and extent of microbial associations and ultrastructurally-resolved architecture of communities is lacking.

Direct sequencing of DNA extracted from natural microbial communities (metagenomics) provides a route to phylogenetic and functional insight without the requirement for laboratory cultivation or isolation. In some instances, the approach can yield near-complete and even complete genomes (Albertsen et al., 2013; Sharon et al., 2013; references therein). These genomes provide a context for functional information from cultivation-independent transcriptomic, proteomic, or metabolomic measurements. However, the utility of this approach is limited by genes and proteins of unknown function, which remain a massive knowledge gap in biology. In some cases ~50% of proteins in organisms from lineages lacking cultivated representatives have no functional prediction (e.g., Kantor et al., 2013). Some of these proteins may be components of biochemically unknown ultrafine structures so far undetected in cultivated microorganisms or isolated organisms. Detection of such features by imaging methods provides a starting point for subsequent targeted investigations to uncover new structural features, appendages, or organelles. Even for structures that can be predicted (e.g., pili and flagella), imaging methods provide information that cannot be deduced from sequence information or expression assays (e.g., the distribution in cells or on cell surfaces, and certain structural characteristics).

Most current image data focuses on a subset of isolates, thus is restricted to a very small proportion of all microorganisms in nature. The effort to visually characterize and ultrastructurally describe microbes in their near intact, physiologically relevant states, within their natural communities has barely begun. Traditional fixation and dehydration methods cause significant artifacts that can be eliminated by using cryogenic TEM (cryo-TEM; see review by Milne and Subramaniam, 2009). Frozen hydrated samples, which are not stained, are imaged using low-dose techniques to minimize radiation damage and preserve structures in a “near native” state. Cryo-TEM provides two-dimensional (2D) images, including fairly high-dose, high signal-to-noise ratio views of selected areas. Cryogenic electron tomography provides three-dimensional (3D) ultrastructural information regarding cell organization and associations with inorganic phases at a resolution up to ~4 nm (Comolli et al., 2009, 2011; Luef et al., 2012). Morphology and ultrastructure are the physical scaffolds underlying and enabling the molecular physiology of the individual populations as well as interrelationships between species in communities. Linking metagenomics, metaproteomics, and metabolomics information with near intact morphology, ultrastructure, and highly resolved spatial interactions and networks remains a grand challenge that must be solved if we want to understand microorganisms in natural context.

Acid mine drainage (AMD) biofilm microbial communities have provided a model system for our efforts to integrate the techniques of cryogenic sample preparation and cryo-transmission electron microscopy (cryo-TEM) and with “omics” data (Supplementary Information; Comolli et al., 2009; Baker et al., 2010; Yelton et al., 2013). Communities are dominated by Leptospirillum bacteria (related to *Leptospirillum ferriphilum* and *Leptospirillum ferrodiazotrophum*), with lower abundances of archaea, mostly from the Thermoplasmatales lineage. Species abundances vary. Relative to other microbial ecosystems, AMD communities are tractable for integrated “omics” and microscopy analysis because they are dominated by a relatively small number of different organism types. Extensive prior metagenomic analyses have documented the presence of novel acidophilic nanoarchaea (ARMAN; Baker et al., 2006, 2010) and Thermoplasmatales lineage archaea (Edwards et al., 2000; Yelton et al., 2013), in association with abundant Nitrospirae phylum (*Leptospirillum* spp., Tyson et al., 2004; Simmons et al., 2008; Aliaga-Goltsman et al., 2009) and less abundant bacteria. See also the review (Baker and Banfield, 2003) for community composition. 3D reconstructions of archaeal ARMAN-lineage cells have revealed intriguing features such as very low ribosome copy numbers and a completely novel cytoplasmic tubular structure (Comolli et al., 2009). These structures may be analogous to those used by bacteria, as discussed by Dubey and Ben-Yehuda (2011), enabling the transfer of macromolecules between individuals. Other studies of AMD biofilms uncovered direct cytoplasmic interaction between ARMAN and Thermoplasmatales lineage archaea, hinting at a solution to the mystery of how the ARMAN, with tiny genomes apparently lacking genes for many core biosynthetic pathways, survive (Baker et al., 2010). Certain Thermoplasmatales lineage archaea cells are decorated with pili, flagella, and S-layers. These features have been linked with genomic data to discriminate between closely related clades and spatially locate cells within communities (Yelton et al., 2013). While the presence of these ultrastructural features implies the expression of corresponding genes, their absence does not provide any conclusion; not all genes are express all the time.

Recently, metagenomic studies have opened new windows on microbial diversity, microbial roles in element cycling and responses to changing environmental conditions. Notably, many organisms very recently described by these methods are predicted to lack many (in some cases, most) core biosynthetic pathways (e.g., members of candidate phyla OD1, OP11, SR1, TM7, BD1-5; Wrighton et al., 2012; Albertsen et al., 2013; Campbell et al., 2013; Kantor et al., 2013), raising the possibility that they are symbionts or parasites as e.g., *Nanoarchaeum equitans* and *Ignicoccus hospitalis* (Jahn et al., 2008). Thus, it is important to pair functional predictions for individual organisms with information about organism associations and interdependencies to understand how microorganisms mediate biogeochemical processes. An early example of possibly key interdependencies is the discovery of archaeal/bacterial strings-of-pearls communities (Rudolph et al., 2001; Moissl et al., 2002). However, no high-resolution, intact ultrastructural studies, nor linked metagenomics, metaproteomics, and metabolomics studies are yet available. Few methods have the ability to resolve microbial cells with sufficient detail to distinguish them from each other and to provide evidence for direct interconnections. Here, we extend our prior studies of AMD archaea, using cryo-TEM to provide further evidence for a fascinating, yet currently almost uninterpretable variety of ultrastructural features in uncultivated organisms. The findings underscore major gaps in our understanding of microbial biology, microbial community functioning, and by inference, microbial evolution. Fully understanding the role of these inter-species interconnections in providing metabolic complementarities across whole microbial communities is central to understanding how they modulate their adaptive and evolutionary responses to environmental pressures.

## Results

**Figure 1:**
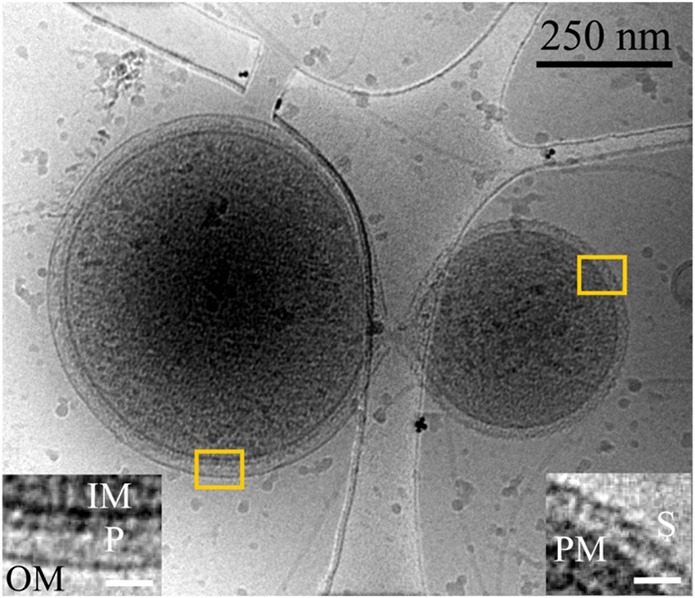
**Inter-species interaction between ARMAN and a *Thermoplasmatal* cell through a cytoplasmic bridge or synapse-like connetion**. 2D cryo-TEM projection showing a “synapse-like” structural connection between an ARMAN cell, left (gram negative-like cell wall), and a *Thermoplasma* “bud” with an S-layer-like outer surface (see also Figure S3; Huber and Stetter, 2006). Insets show a magnified view of the regions within the yellow box, left and right respectively. Left inset: IM, OM, P are inner membrane, outer membrane, and periplasmic space respectively. Right inset: PM and S are the plasma membrane and S-layer respectively. The white scale bars are 25 nm.

**Figure 2:**
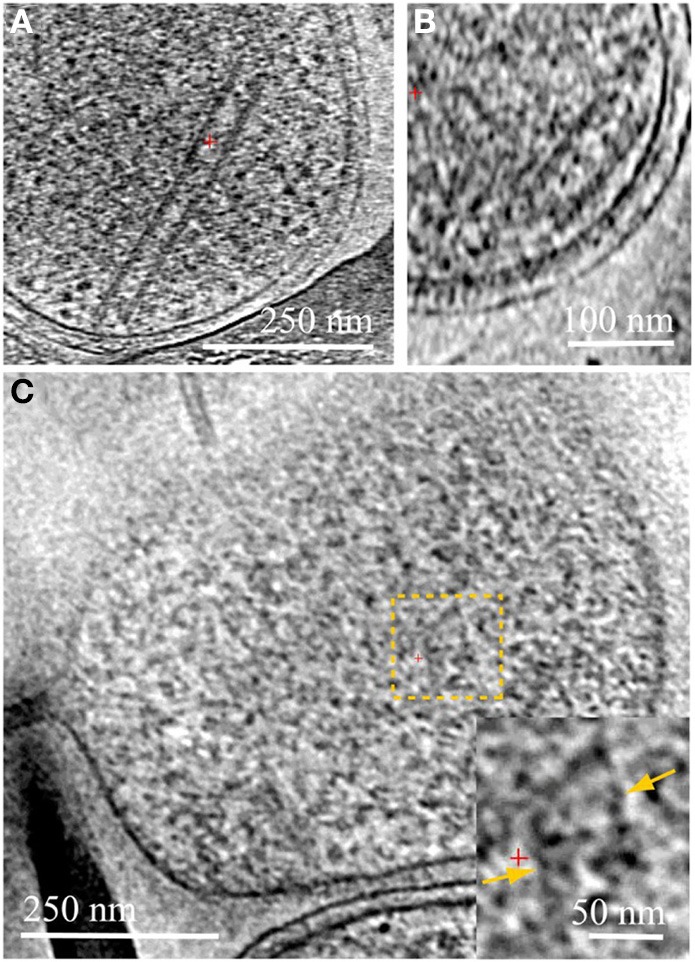
**Cytoplasmic structures in ARMAN and in *Thermoplasmatales***. Computational slices through 3D cryo-ET reconstructions of two ARMAN cells **(A,B)** and a *Thermoplasma* lineage cell **(C)**. The tubular structure in **(A)** is one of the longest observed, ~400 nm. In **(B)** the short tube is adjacent to the cell wall but we do not resolve any mechanistic association between tube and cell wall. These tubular structures are filled with a density typical of the cytoplasmic space including high-contrast elements. *Thermoplasmatales* lineage archaea can also have cytoplasmic structures in the same range of widths, such as in the cytoplasmic region within the dashed yellow box; a magnified view is shown in the inset, with the parallel boundaries indicated by yellow arrows. See also Figures S2–S6 and Movies 1–4.

**Figure 3:**
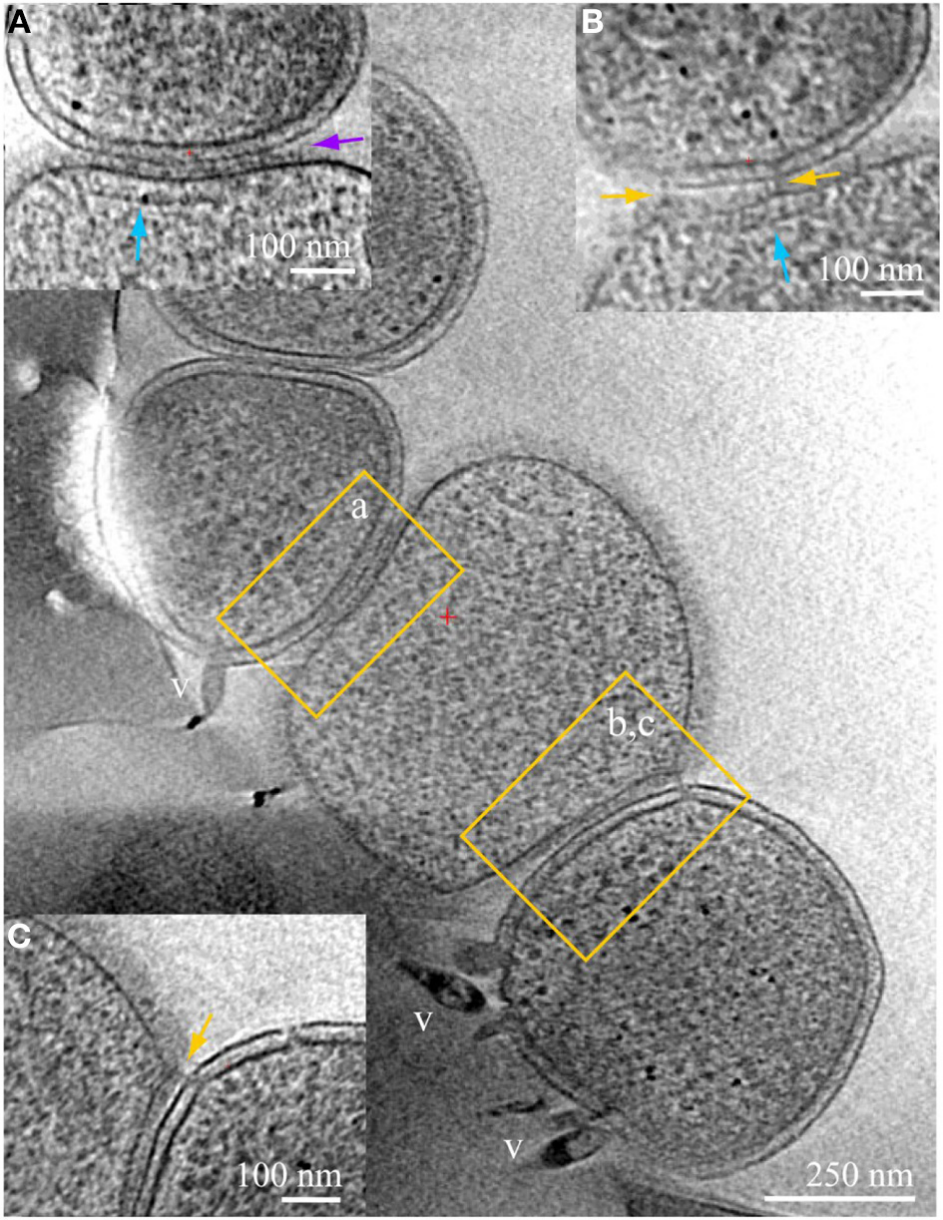
**ARMAN—*Thermoplasmatales* inter-species associations and viral infection processes**. Slices from a 3D cryo-ET reconstruction of a colony of cells showing contacts and associations between a *Thermoplasmatal* lineage cell (center, no cell wall) and two ARMAN cells infected with viruses (gram-negative like cell wall, top left, and bottom right). Insets represent slices at different orientations, of the regions within yellow boxes in the main z-slice. In inset **(A)** the cell surfaces are in tight contact, with a distinct line of density at the interface pointed by the purple arrow. *Thermoplasma* lineage archaea cells engaged in this type of association typically have a cytoplasmic band of density parallel to the plasma membrane, indicated here by the vertical blue arrow. These features resemble, to some extent, bacterial chemotaxis apparatus (Milne and Subramaniam, 2009), but AMD *Thermoplasmatales* lineage archaea have no identified chemotaxis genes. Inset **(B)** is a slice at an orientation that exactly contains a filamentous connection between the cells that clearly crosses the cell wall; the same band of density as in **(B)** is indicated by a blue arrow. Inset **(C)** is another slice of the same region, at different coordinates (z-height and angles), with pili-like connections through the cell wall. The ARMAN cells involved in the interactions shown in insets **(A–C)** are infected by rod-shape and lemon shaped viruses some of which are clear and labeled “v.” See also Figures S2–S6 and Movie 4.

The AMD microbial community samples contain *Thermoplasmatales* lineage archaea referred to as Aplasma, Eplasma, Gplasma, Ferroplasmas Fer1 and Fer2 [related to previously described *Ferroplasma* and *Thermoplasma acidophilum* (Golyshina and Timmis, 2005)]; Iplasma from a sibling lineage to the *Thermoplasmatales* (Yelton et al., 2013); as well as ARMAN nanoarchaea (Baker et al., 2006). Whole ultrastructural characterization of ARMAN nanoarchaea was reported previously (Comolli et al., 2009); their cell walls are smooth and uniform in thickness (~30 ± 2 nm wide), with conspicuous inner and outer membranes (IM, OM) and no S-layer. The periplasmic space is studded with ~5–8 nm diameter high contrast features in close proximity to the IM and with physical connections through the IM into the cytoplasm (Figure 1; Comolli et al., 2009). Distinguishing *Thermoplasmatales* lineage archaea from the other archaea is straightforward on the basis of size and irregular and pleomorphic morphology and absence of cell wall (Yasuda et al., 1995; Golyshina and Timmis, 2005; Yelton et al., 2013 and references therein; Figures S2–S6). *Thermoplasmatales* lineage archaea cells are generally bounded by a single membrane; all of the AMD plasma genomes (except Fer 1) have putative S-layer genes, and also genes potentially involved in archaeal S-layer protein N-glycosylation (Figures 2, 3; Figures S2–S6; Movies 1, 2; Yelton et al., 2013). Most *Thermoplasmatales* lineage archaea lack flagella genes, except for A or E plasma, which have complete sets of flagella genes.

We surveyed the associations between ARMAN and *Thermoplasmatales* lineage archaea cells and detected a series of repeated patterns of physical associations. A striking physical interaction occurs via direct communication between cytoplasmic spaces through a “synapse-like” structure as the one shown in Figure 1. A protrusion in the cell wall of ARMAN with a hole approximately 30 nm in diameter is coupled to a hole through the S-layer and plasma membrane of the *Thermoplasma* lineage cell, or most likely a “bud” separated from a larger cell, providing a direct connection between the cytoplasmic spaces. The outer membrane of the cells interacting with ARMAN is typically covered with a 5–7 nm thick periodic layer inferred to be an S-layer. *Thermoplasma* lineage cell typically form “buds” which are released from the original cell (Huber and Stetter, 2006). See also Figure S3.

We consistently observe large tubular structures within ARMAN (Comolli et al., 2009; Figure 2; Movie 3). We have also observed structures in the cytoplasmic space of *Thermoplasmatales* lineage archaea (Figure 2). However, we have not resolved whether they are involved in interspecies connections.

Physical contacts are commonly observed, often involving more than two cells (Figure 3). Large areas of cell surfaces are in very close contact in some instances. We detected Velcro-like or zipped surface densities at some interfaces, as in inset (a). The same surfaces in contact are also linked by pili-like fibers, as in inset (b), and by appendages connecting *Thermoplasmatales* lineage archaea that through ARMAN cell wall, as in inset (c).

**Figure 4:**
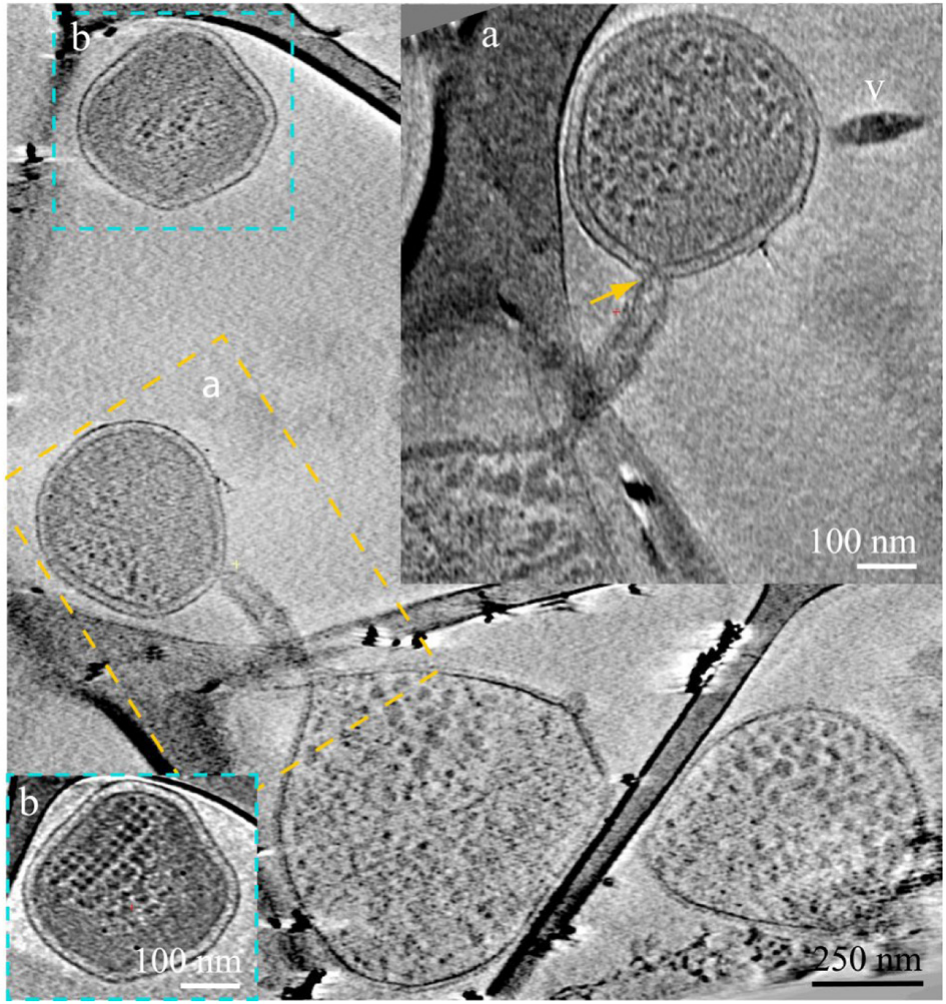
**ARMAN—*Thermoplasmatales* inter-species associations and viral infection processes**. Slices from a 3D cryo-ET reconstruction. A *Thermoplasmatal* cell and an ARMAN cell—enclosed within the dash yellow box, are connected through a remarkable tubular appendage; the region within the dash yellow box is magnified as inset **(A)** in a slice at a different angle. The “arm-shape” connection extends the *Thermoplasmatal* cytoplasmic compartment and punctures the cell wall of ARMAN establishing an inter-species cytoplasmic bridge (yellow arrow). An elongated virus is partially in plane to the right (v). The cell within the blue box is shown in inset **(B)** in a slice at a different angle. The very regular ladder-like series of high contrast elements are putative polysomes. These cells are of an unknown species and are often found connected to *Thermoplasmatal* cells through pili, as shown in Figure 6 with more detail.

**Figure 5:**
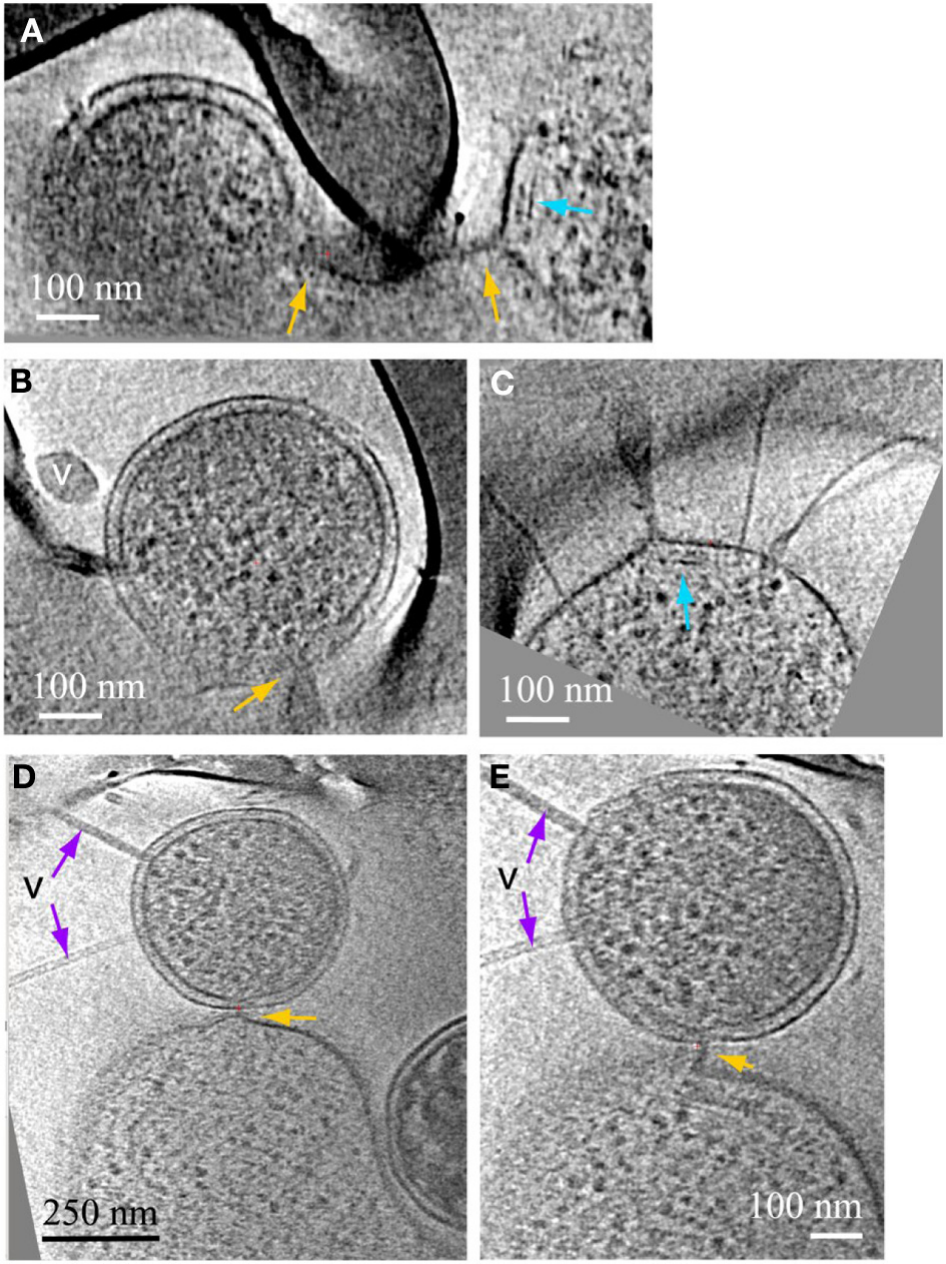
**ARMAN—*Thermoplasmatales* inter-species associations and viral infection processes**. Slices from a 3D cryo-ET reconstruction. **(A–C)** are computational slices at different orientations through an ARMAN and a *Thermoplasmatales* lineage archaea cell connected through a cytoplasmic bridge. In **(A)** the cytoplasmic bridge is directly under a grid bar with ARMAN to the left and the plasma cell to the right respectively. The blue arrow points to a band, within the cytoplasmic space, adjacent to the connection. **(B)** The ARMAN cell in **(A)**; the yellow arrow points to the cytoplasmic side of the connection; the ARMAN cell is infected by a virus (v) of a different morphotype than in Figure 4. **(C)** The *Thermoplasmatales* lineage cell in **(A)**; the putative flagella indicates the plasma cell is either **(A** or **E)**; for instance, see above and to the right of the feature indicated with the blue arrow. **(D,E)** Are slices through a different type of ARMAN (top)-*Thermoplasmatal* (bottom) lineage archaea association. The ARMAN cell, top, is infected by rod-shape viruses (v) indicated by purple arrows and the connection (yellow arrow) is a short and thick line of density.

Another type of prevalent association is established over sub-micron scale distances via a long tubular structure formed by a *Thermoplasmatales* lineage archaea cell, as in Figures 4, 5A–C. These tubular structures have a diameter equal or larger than the synapse of Figure 1, and they completely penetrate through the cell walls of ARMAN. In some cases, as in Figure 5, we have identified flagella on the *Thermoplasmatales* lineage archaea, narrowing the possible lineages to either A or E plasmas (based on genome prediction; Yelton et al., 2013). It is thus plausible that each lineage of *Thermoplasmatales* uses a specific mode of interaction.

**Figure 6:**
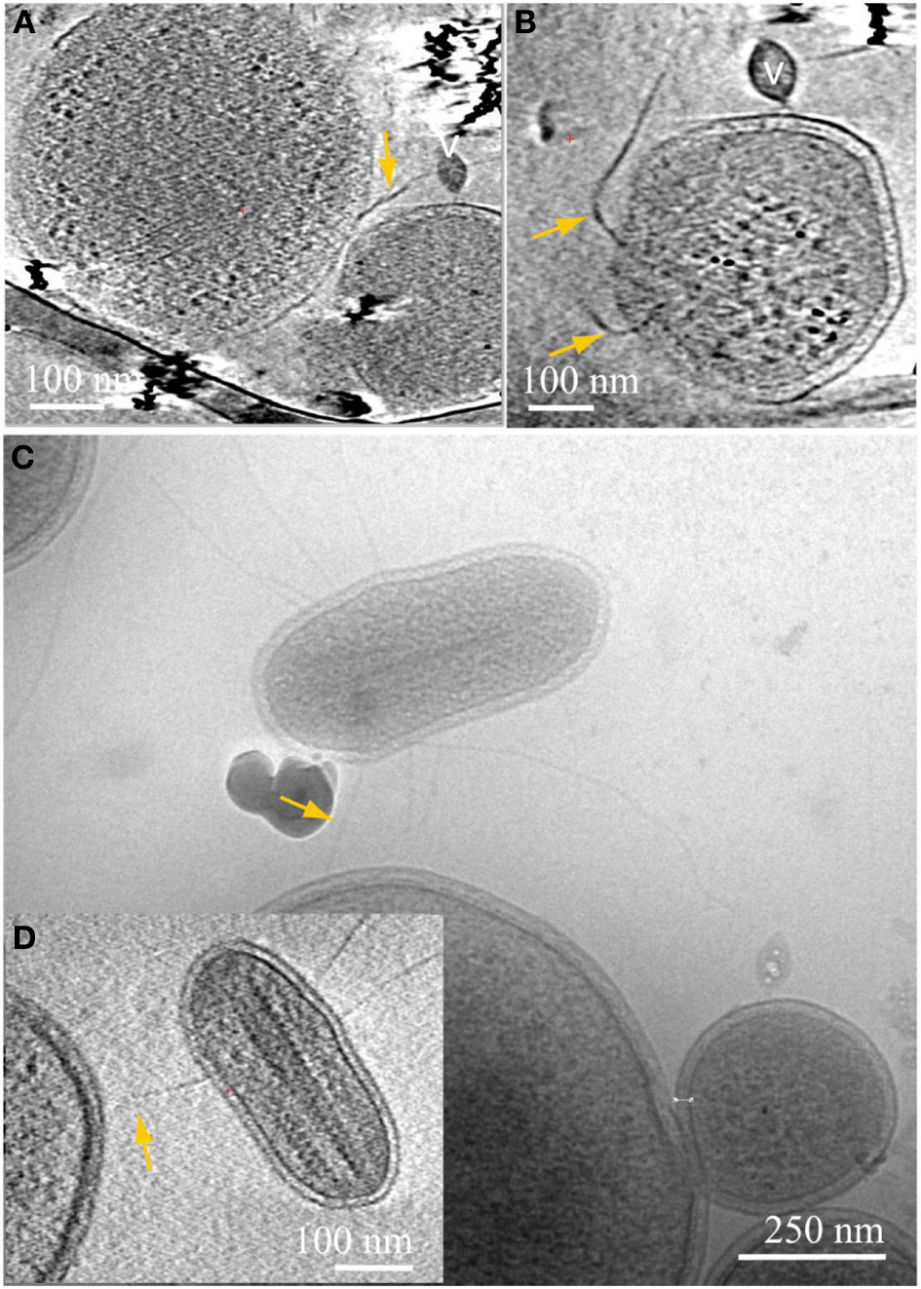
**Inter-species associations through pili**. Computational slices through 3D cryo-ET reconstructions of an archaea **(A)**, connected to the center of an ARMAN cell, bottom right, through thick pili; the ARMAN cell sliced through a different angle is magnified in **(B)**. The complete trajectory can be followed in 3D but falls out of plane in these sections. **(C)** 2D cryo-TEM projection and inset **(D)** slice through 3D cryo-ET reconstruction of an unknown archaeal cell with large putative polysomes, as in inset **(B)** of Figure 4. The microorganism is connected through putative pili indicated by yellow arrows to a large cell. See also Movies 5, 6.

We observed a variety of other intriguing, but still enigmatic associations. For example, some ARMAN cells are penetrated by long, thick pili that extend from other organisms (Figures 6A–C). Other very small, compact cells of unknown identity, studded with cytoplasmic ladder-like structures (hypothetically polysomes, Figure 6), were observed to establish connections with much larger cells (Figures 6C,D).

**Figure 7:**
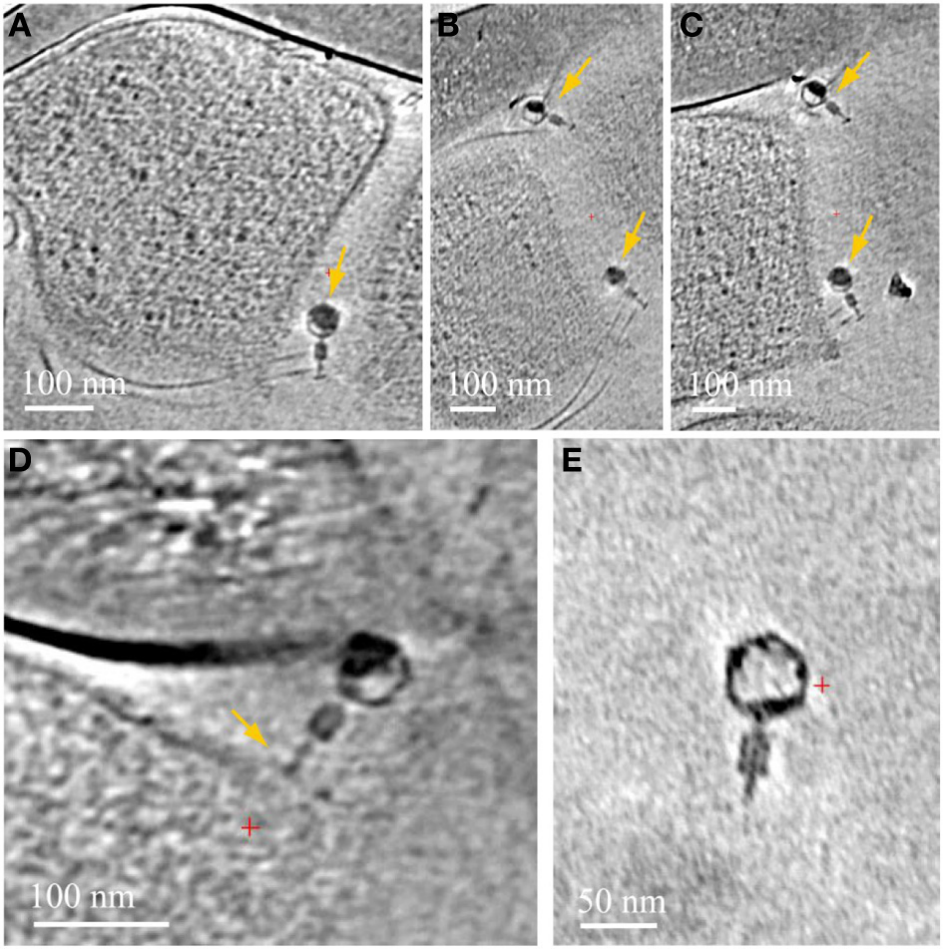
**Associations between viruses and *Thermoplasmatales* pili**. Computational slices through 3D cryo-ET reconstructions of *Thermoplasmatales* lineage archaea associated with viruses. **(A)** A virus attached to pili, close to the cell surface; **(B,C)** two slices at different heights and angles through the same reconstruction as in **(A)**, with two viruses attached to pili, yellow arrows. Pili are very thin and a series of slices are needed to capture them, and the viruses, in-plane of the slices respectively. The top and bottom viruses are shown in Supplementary Information Movies 7, 8 respectively. **(D)** a virus attached to a cell surface. **(E)** A free virus.

We very frequently detected viruses infecting the ARMAN cells, in general of two different morphotypes, based on distinct virus particle morphology. In some cases we observe a thick surface density layer on the *Thermoplasmatales* lineage archaea cells that are associated with ARMAN cells infected by viruses, but no regular S-layers (Figure 3). *Thermoplasmatales* lineage archaea cells also often had surface-attached viral particles. The viruses that infect ARMAN and the viruses that infect *Thermoplasmatales* lineage archaea are morphologically distinct. The Thermoplasmatales-related cells also had viruses attached to their putative pili (Figure 7).

## Discussion

Because thin, small samples of were obtained directly from the natural environment and instantly frozen as fragments of biofilm, the cryo-TEM imaging and 3D cryo-ET reconstructions show us microorganisms in their mutual relationships with the intact ultrastructure, in the full context of their mutual relationships. We detected a variety of physical connections amongst uncultivated archaeal cells, include synapse-like connections, connections between cytoplasmic spaces through tubular structures, needle-like penetration of ARMAN's cell wall by *Thermoplasmatales* lineage archaea (Comolli et al., 2009), Velcro-like contacts and string-like attachments between their cell boundaries. These may enable cells from distinct phyla to share small molecules as well as larger proteins. Alternatively, rather than symbiotic, the association may be predatory. All *Thermoplasmatales* lineage archaea except for Fer1 have S-layer genes and most lack flagella genes; cells in Figures 5, 6 can only be A or E plasma, which have complete sets of flagella genes.

ARMAN cells are typically found in AMD microbial communities that include *Thermoplasmatales* lineage archaea cells. Given the tiny size of the ARMAN genome (~1 Mb), and it's apparently lack of many biosynthetic pathways, some level of dependence of ARMAN on the larger archaea seems likely. Systems-biology tools are revealing networks of interacting microorganisms (e.g., Fuhrman, 2009). A pivotal question in evolutionary biology is the emergence of cooperative traits and their sustainment in the presence of free-riders. In order to understand how cooperation can emerge and be maintained, Cremer et al. proposed various strategies of spatial distribution within biofilms (Cremer et al., 2012). They found that “demographic noise during population bottlenecks creates a broad distribution in the relative abundance of cooperators and free riders. The growth advantage of more cooperative subpopulations implies an asymmetric amplification of fluctuations and possibly yields to an increase of cooperation in the whole population (group-growth mechanism).” Their analysis shows that this can enable a single cooperative mutant to spread in the population which then, mediated by the dynamics, reaches a stationary state with coexisting cooperators and free-riders (Cremer et al., 2012). It seems unlikely that ARMAN behave as free-riders, since *Thermoplasmatales* lineage cells create many of the physical connections; at the same time, ARMAN likely depends on other archaea as discussed above. The ultrastructural survey presented here establishes physical architectures underlying networks and enabling metabolic exchange between microorganisms. A new capability will be needed to prove, with subcellular resolution, the exact molecular nature of these interactions.

The capacity for recognition, which must be essential for formation of the associations observed here, is likely embedded in the macromolecular surface assemblies, pili, and other sensory apparatus. Tackling questions related to interspecies interactions is important for understanding the environmentally and medically important process of biofilm formation. There, a cooperating collective of microorganisms runs the risk to be undermined by members that do not contribute to the metabolically costly task of biofilm polymer formation. It is notable that the findings of the current study center on interspecies interactions that do not produce a “common good” (such as the constituents of the biofilm architecture). Rather, all associations are cell-to-cell. An implication of the observed associations is that the microorganisms involved almost certainly co-evolve.

The tendency of certain archaeal cells to establish interspecies interactions has gone largely undetected until recently. *Pyroccocus furiosis* and *Pyrodictium* have been shown to connect through flagella and cannulae respectively (Nickell et al., 2003; Näther et al., 2006). The two archaea *Ignicoccus hospitalis* and *Nanoarchaeum equitans* form an intimate association, the character of which is not yet fully understood (Jahn et al., 2008). Cryo-TEM show that at least two modes of cell-cell interactions exist: (i) the two cells are interconnected via thin fibers; and (ii) the two cell surfaces are in direct contact with each other (Junglas et al., 2008). Although *N. equitans* obtains lipids and aminoacids from its host, no direct cytoplasmic connection has been observed (Jahn et al., 2008). It is often the case with archaea, extremophiles, and intact microbial communities that singular novel observations precede our ability to provide complete functional interpretations; another intriguing example in this context is the structural appendage named “hamus” (Moissl et al., 2005), involved in archaeal cells adhesion and anchoring.

Dubey and Ben-Yehuda (2011) showed that nanotubes can form between *Bacillus subtilis* cells, enabling bacteria to transfer proteins and genetic material between them. They also show the capacity of *Bacillus subtilis* to transfer proteins to *Staphylococcus aureus* and to *Escherichia coli*, different species of bacteria. While this was shown in *in-vitro* experiments these results clearly suggest the potential for this type of communication is present in biofilms, enabling bacteria to adapt to adverse conditions collectively.

Archaea can have unique cell envelopes, lacking any component outside the cytoplasmic membrane other than surface proteins, S-layers, or glycoproteins (Albers and Meyer, 2011). One of the possible consequences or attributes of the unique archaeal cell wall structure may be this ability to recognize interacting partners, to readily and actively deform and extend through space, crossing through occluding objects in biofilms, and to puncture into the cytoplasmic space of interacting partners. These structures suggest an active mechanism of reaching out, searching and penetrating ARMAN cells by *Thermoplasmatales* lineage archaea.

We observed many archaeal pili associated with viruses. Laboratory work has shown the capacity of bacteriophage to use bacterial pili as an “alternative mechanism” available for infection (Guerrero-Ferreira et al., 2011). Our cryo-TEM data suggest this interaction may also occur between archaea and their viruses. Pili have been known to mediate in cell-to-cell connections, genetic recombination, and attachments. Yet we find the extent of the physical contacts established by pili, and the range of inter-species bridged by them, including viral-microbes interaction, to warrant a new examination from an ecological and evolutionary perspective. Pili may guide them toward the host cell or provide a direct access route for viral DNA. Alternatively, pili may trap viral particles and thus serve as part of the viral defense mechanism of the host cells.

The findings of the current study reinforce the idea that our limited ability to cultivate microorganisms, and thus to understand them, rests with interdependence on other organisms. The study of microbes in their intact natural context enables evidence for such associations to be detected. Ultrastructural characterization of uncultivated cells from novel lineages also reveals the existence of features (possibly organelles) whose functions and biosynthetic capacities are unknown.

## Methods

### Cryo-EM specimen preparation

For cryo-transmission electron microscope characterization, aliquots of 5 μl were taken directly from fresh biofilm samples and placed onto lacey carbon grids (Ted Pella 01881) that were pre-treated by glow-discharge. The support grids were pre-loaded with 10 nm colloidal gold particles. The Formvar support was not removed from the lacey carbon. The grids were manually blotted and plunged into liquid ethane by a compressed air piston, then stored in liquid nitrogen. For electron microscopy at room temperature, 5 μl aliquots were placed on glow-discharged Formvar carbon coated grids (Ted Pella 01811), then the grids were blotted and air dried. Samples were carefully transported to the laboratory bench for cryo-plunging within the same day; samples were also cryo-plunged inside the IMM, as previously described (Supplementary Information; Comolli et al., 2011; Knierim et al., 2011). For more details see Comolli et al. (2009, 2011); Baker et al. (2010); Yelton et al. (2013). Supplementary Information Figures S1–S6 provide a useful survey from sampling to cryo-TEM images of complex regions with healthy and dead cells.

### Cryo-electron tomographic imaging

Samples were transfered from the liquid nitrogen storage to the electron microscope sample holder for data acquisition. Images were acquired on a JEOL–3100 transmission electron microscope equipped a FEG electron source operating at 300 kV, an Omega energy filter, a Gatan 795 2 × 2 K CCD camera, and cryo-transfer stage. The stage was cooled using liquid nitrogen to 80 K.

In order to have a statistically relevant survey of cell sizes and morphologies, over 800 images or 2D projections were recorded using magnifications of 36, 30, and 25 Kx at the CCD, giving a pixel size of 0.83, 1.0, or 1.2 nm at the specimen, respectively. Underfocus values ranged between 8 ± 0.5 μm and 14 ± 0.5 μm, and energy filter widths were typically around 22 ± 2 eV. The survey of the grids and the selection of suitable targets for tilt series acquisition were done in low dose diffraction mode through the acquisition of hundreds of images. For a discussion on electron dose and radiation damage see (Knierim et al., 2011).

Tomographic tilt series were acquired under low dose conditions, typically over an angular range between +65 and −65°, ± 5° with increments of 1 or 2°. Between 70 and 124 images were recorded for each series. A total of 69 3D data sets were obtained: 11 tilt series were acquired manually with the program Digital Micrograph (Gatan, Inc.), and 58 tilt series were acquired semi-automatically with the program Serial-EM (http://bio3d.colorado.edu/) adapted to JEOL microscopes. All images were recorded using a magnification of 36, 30, or 25 Kx at the CCD giving a pixel size of 0.833, 1.0, or 1.2 nm at the specimen respectively. Underfocus values ranged between 9 ± 0.5 μm to 16 ± 0.5 μm, depending on the goal of the data set, and energy filter widths ranged between 22 to 28 eV, also depending on the data set. For all data sets the maximum dose used per complete tilt series was ~150 e-/Å^2^, with typical values of ~100 e-/Å^2^.

It is important to clarify that cryo-TEM imaging is limited to a thickness of ~750 nm or less; while ARMAN and small bacteria fall well within this range, larger microorganisms can exceed this limit; complex, rich areas of biofilms are certainly thicker and thus not accessible to this methodology. Although in the current sampling we have observed a percentage of ~30 % ARMAN involved in interactions and inter-connections, only a large scale effort using different technologies can provide a reliable estimate. The signal-to-noise for a fixed dose is in inverse relationship to sample thickness; consequently our resolution is lower for larger microorganisms and complex regions of biofilms. Many of these limitations could be overcome in the future with cryo-sectioning. It would currently not be scientifically rigorous to extrapolate from direct cryo-TEM of sufficiently transparent, microscopic areas of biofilms, to the whole system of biofilms covering geographical scales. Instead, these observations and the linking between genomics and cryo-TEM advanced in this manuscript underlie the need to leverage a multidisciplinary approach to these questions (the core motivation of this Frontiers Special Topic). For instance, a large scale effort linking cryo-TEM, FIB sections and cryo-TEM, FIB SEM milling, plastic sections TEM, light microscopy and correlative FISH-cryo-TEM would be a natural next step.

### Image processing and analyses

All tomographic reconstructions were obtained with the program Imod (http://bio3d.colorado.edu/). The program ImageJ (NIH, http://rsb.info.nih.gov/ij/) was used for analysis of the two-dimensional image projections. Volume rendering and image analysis of tomographic reconstructions was done using the program VisIt (http://www.llnl.gov/visit). All movies were done with the package ffmpeg (www.ffmpeg.org).

## Author contributions

Study conception and experimental design: Luis R. Comolli and Jill F. Banfield. Cryo-TEM data acquisition, processing, and analysis: Luis R. Comolli. Linking “Omics” with imaging data: Jill F. Banfield. Integrated data survey and analysis: Jill F. Banfield and Luis R. Comolli. Writing and editing of the manuscript: Luis R. Comolli and Jill F. Banfield.

### Conflict of interest statement

The authors declare that the research was conducted in the absence of any commercial or financial relationships that could be construed as a potential conflict of interest.

## Supplementary material

Movie 1 **Compressed view of the cryo-ET reconstruction of a *Thermoplasmatales* cell; a high quality slice through the 3D reconstruction is shown in Figure S1A**.

Movie 2 **Compressed view of the cryo-ET reconstruction of a *Thermoplasmatales* cell; a high quality slice through the 3D reconstruction is shown in Figure S1B**.

Movie 3 **Volumen-rendered view of a 3D section from a cryo-ET reconstruction of ARMAN cells**. A large tubular structure is seen in the center. See also main text Figure 2.

Movie 4 ***Thermoplasmatales*, ARMAN, and interconnections**. Compressed view of the cryo-ET reconstruction of associated and inter-connected Tpl and ARMAN shown in Figure 3 of the main manuscript.

Movie 5 **Compressed view of the cryo-ET reconstruction from which the slices shown in Figures 6A,B of were extracted**.

Movie 6 **Compressed view of the cryo-ET reconstruction from which the slice shown in Figure 6D of was extracted**.

Movie 7 **Compressed view of the cryo-ET reconstruction from which the slices shown in Figures 7A–C of were extracted**. The virus is the top virus in those panels.

Movie 8 **Compressed view of the cryo-ET reconstruction from which the slices shown in Figures 7A-C of were extracted**. The virus is the bottom virus in those panels.
