# Supplementary material for: Inter-species interconnections in acid mine drainage microbial communities
Source: Front Microbiol. 2014 Jul 25;5:367. doi: 10.3389/fmicb.2014.00367 (PMC4110969; doi:10.3389/fmicb.2014.00367)
Supplement: Supplementary file 9 [file DataSheet1.DOCX]

**Supplementary Information**

Sampling from AMD biofilms at location UBA Dam, Iron Mine Mountain (IMM), Redding CA, USA. More geographical and topological details have been reported before (e.g. Baker and Banfield, 2003; Tyson et al., 2004; Baker et al., 2006; Baker et al., 2010; and references therein).

Several samples were transported to the laboratory bench, in containers, for cryo-plunging within the same day. On November-02-2010 we cryo-plunged inside the IMM, as previously described (Comolli et al., 2012). In all cases samples were prepared simultaneously for genomics, proteomics, and imaging. However, the “size” or net volume needed for cryo-samples is significantly smaller, at ~ 5 μl per grid, than what is used for the other methods, typically dozens of milliliters or more. The implication is that metagenomics and metaproteomics provide bulk averages at a large scale; cryo-samples may cover a range of microscopic, or local, anisotropies. We thus caution the attempt to extrapolate global or mesoscopic level memberships and architectures from cryo-samples.

**Figure S1**


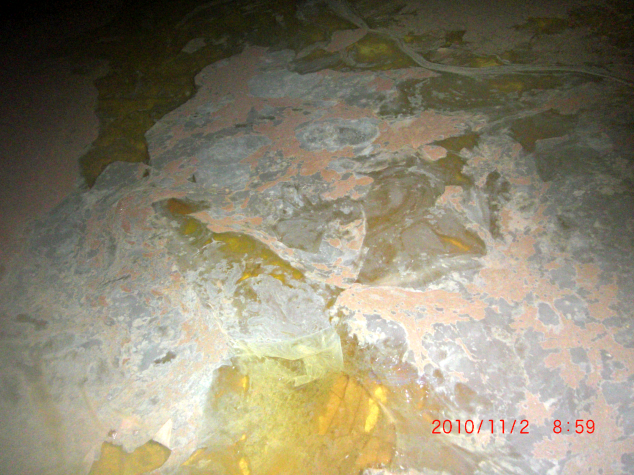

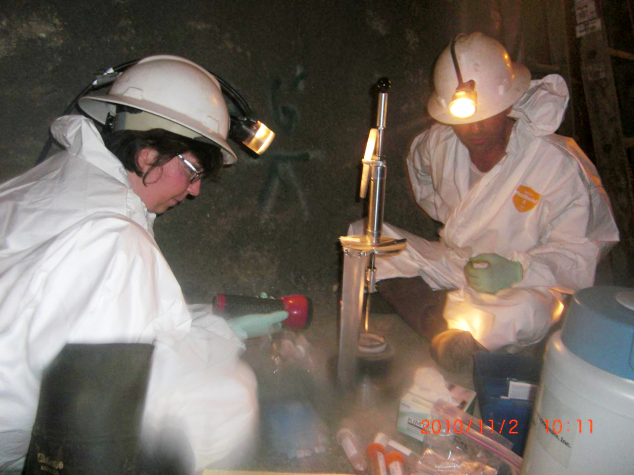


**Sampling in the IMM, Redding, CA.** Left, view of UBA Dam biofilm growing in a subsurface pond inside the mine tunnels. Right, making cryo-samples inside the mine, as reported previously (Comolli et al., 2012).

Cryo-samples require very small amounts of material (one grid is less than ~ 3 mm in diameter on which a drop of ~ 5 μl is deposited prior to blotting and flash-freezing). The proportion of groups of microorganisms and their distribution as found on the scale of cryo-grids should not be directly extrapolated to the whole biofilm. Future work using Nano-Sims and SEM of large areas, correlated with metagenomics, metaproteomics, light microscopy and cryo-TEM will further characterize biofilms at the mesoscopic-level.

**Figure S2**


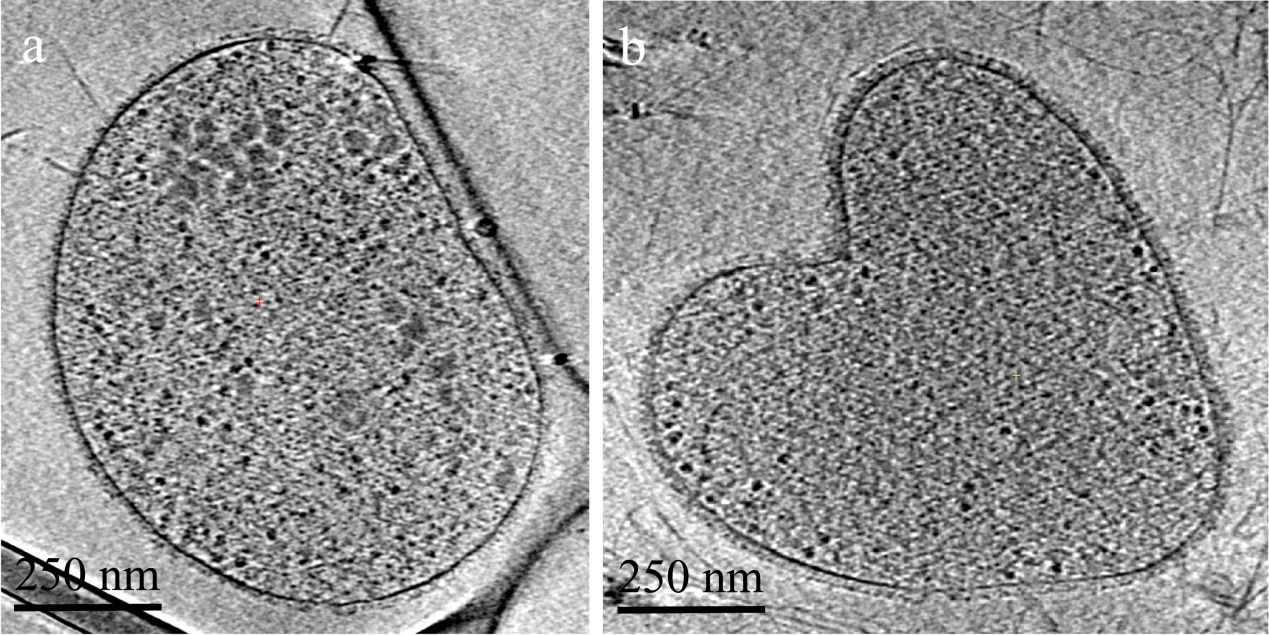


***Thermoplasmatales.*** Computational slices through 3D cryo-ET reconstructions of two *Thermoplasmatales* lineage cells. The cell in a) contains many typical vacuoles, and the plasma membrane is surrounded by a thin outer layer. The cell in b) has the plasma membrane covered by a thicker and more structured outer layer, probably an S-layer. See also Movies 1 and 2 for the compressed, full 3D reconstruction of these same cells respectively.

**Figure S3**


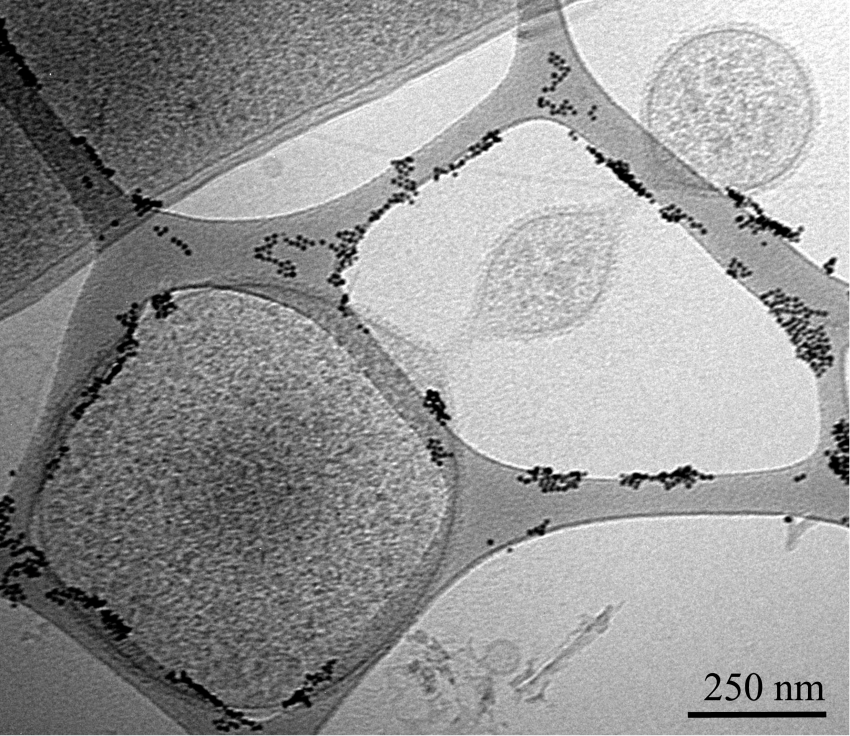


***Thermoplasmatales buds.*** 2D cryo-TEM projection of a *Thermoplasmatales* lineage cell of the same type as in Figure1, forming buds (see also Huber and Stetter, 2006).

**Figure S4**


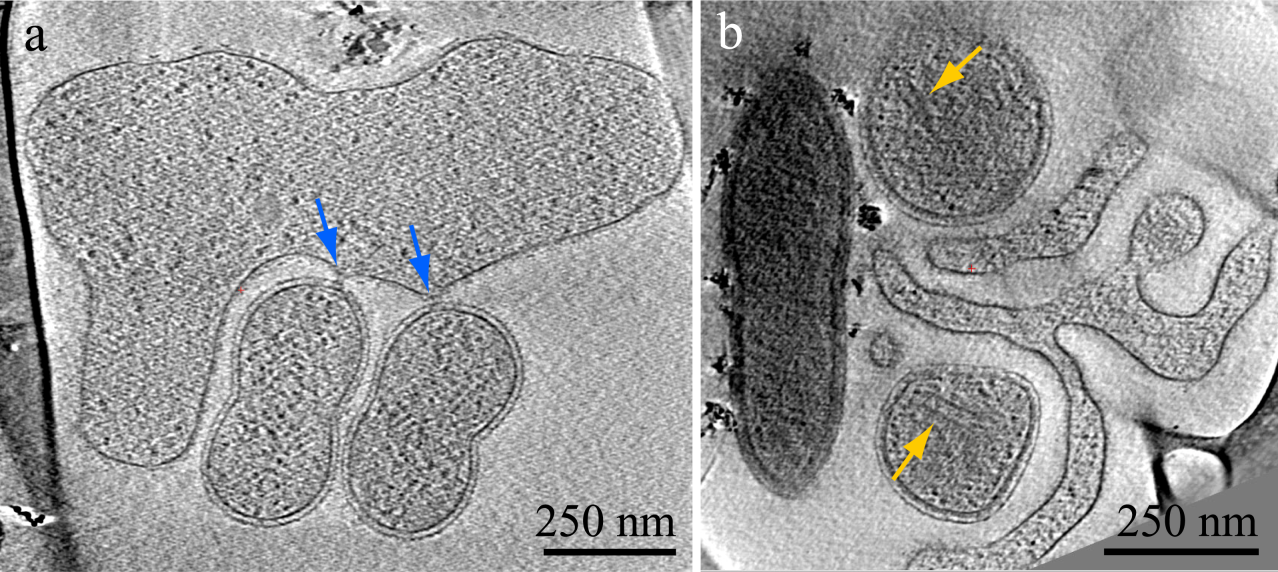


***Thermoplasmatales* and interactions*.*** Computational slices through 3D cryo-ET reconstructions. The large cell in a) is a typical *Thermoplasmatales* cell with no cell wall, bounded by a plasma membrane covered by a thin sheath or layer of organic polymer (typically glycoproteins), and highly irregular, pleomorphic shapes. Smaller cells of a different species on the basis of the cell envelope are physically connected to the large cell –blue arrows. A slice through a reconstruction of a thick region including bacterial and archaeal species shows the potential for intriguing biology but limiting signal-to-noise ratio. Tubular structures within ARMAN cells are indicated by yellow arrows. The labels “Bact” and “Tpl” refer to bacterial and *Thermoplasmatales* cells. The presence or absence of interconnections between the ARMAN and Tpl cells cannot be rigorously assessed due to the thickness.

**Figure S5**


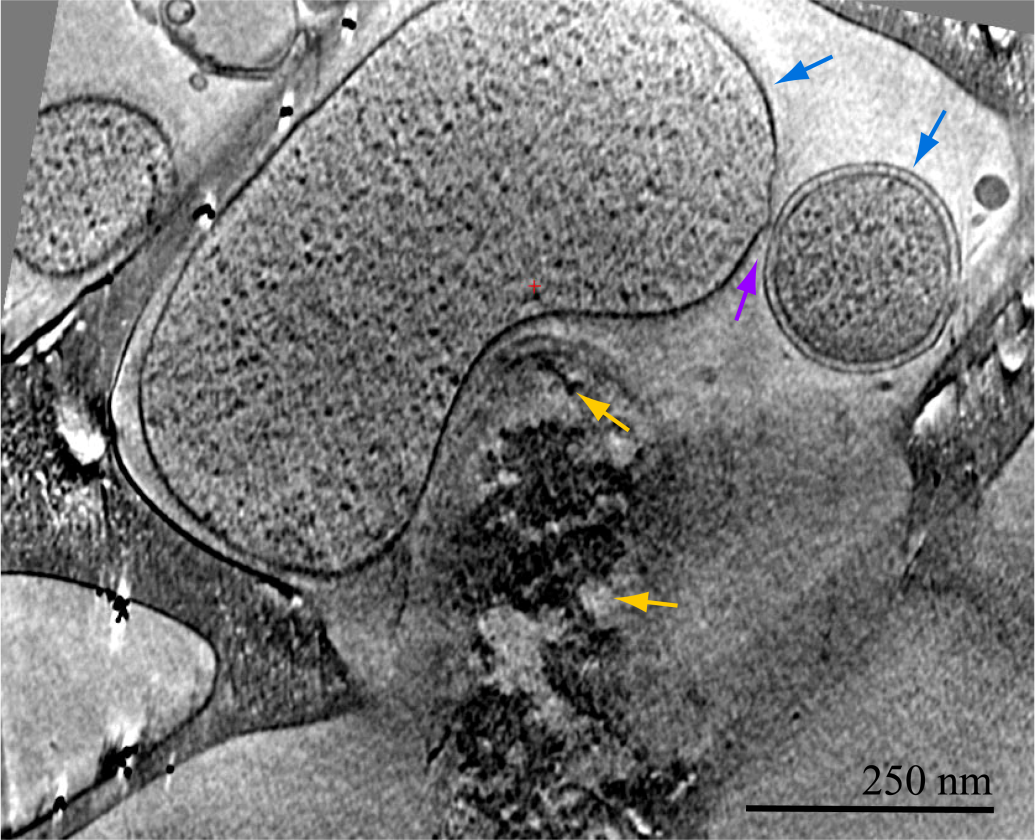


**Healthy and dead cells**. Computational slice through a 3D cryo-ET reconstruction of a thick region with healthy and dead cells, blue and yellow arrows respectively. The two healthy cells are making an interconnection, purple arrow. The large cell is a typical Tpl while the small cell, right side, has an ARMAN architecture.

**Figure S6**


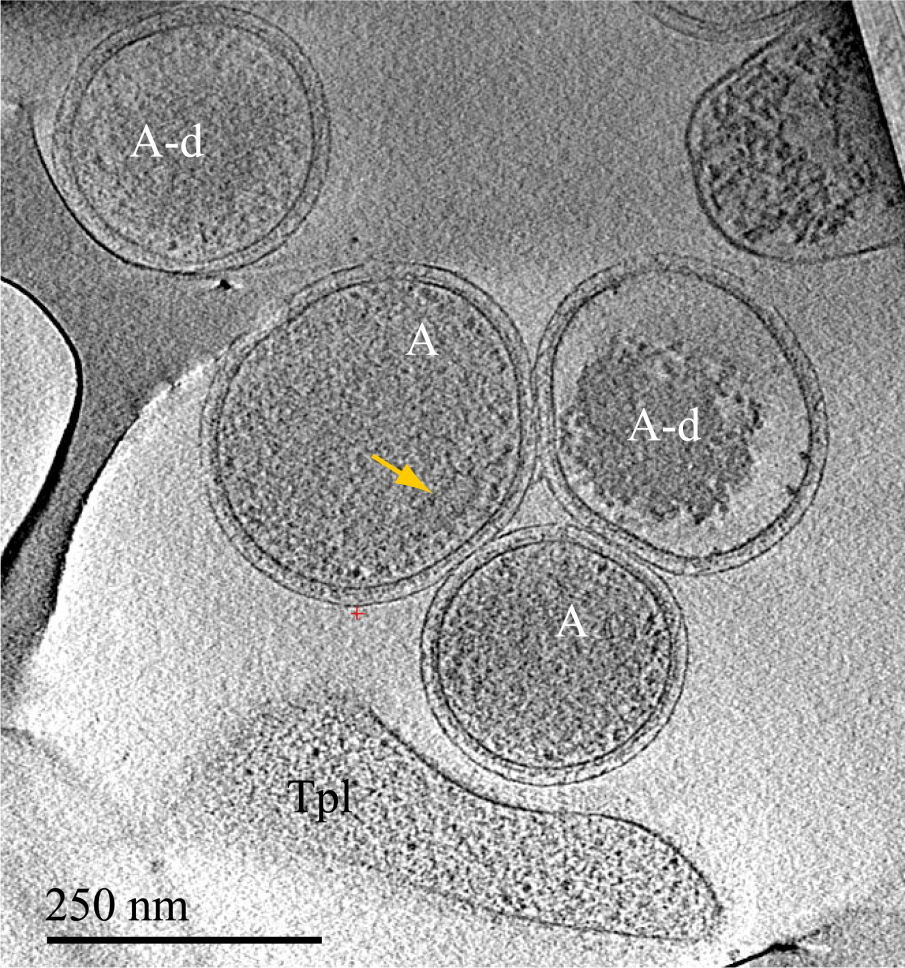


**Healthy and dead cells**. Computational slice through a 3D cryo-ET reconstruction of a thick region with healthy and dead cells. The label “A” indicates healthy ARMAN cells; the yellow arrow points to a tubular structure; “A-d” indicates dead ARMAN cells; “Tpl” indicates *Thermoplasmatales* lineage archaea (part of a pleomorphic cell).
